# Differential effects of white noise in cognitive and perceptual tasks

**DOI:** 10.3389/fpsyg.2015.01639

**Published:** 2015-11-03

**Authors:** Nora A. Herweg, Nico Bunzeck

**Affiliations:** ^1^Department of Systems Neuroscience, University Medical Center Hamburg-EppendorfHamburg, Germany; ^2^Department of Psychology, University of LübeckLübeck, Germany

**Keywords:** stochastic resonance, stochastic facilitation, dopamine, memory, attention

## Abstract

Beneficial effects of noise on higher cognition have recently attracted attention. Hypothesizing an involvement of the mesolimbic dopamine system and its functional interactions with cortical areas, the current study aimed to demonstrate a facilitation of dopamine-dependent attentional and mnemonic functions by externally applying white noise in five behavioral experiments including a total sample of 167 healthy human subjects. During working memory, acoustic white noise impaired accuracy when presented during the maintenance period (Experiments 1–3). In a reward based long-term memory task, white noise accelerated perceptual judgments for scene images during encoding but left subsequent recognition memory unaffected (Experiment 4). In a modified Posner task (Experiment 5), the benefit due to white noise in attentional orienting correlated weakly with reward dependence, a personality trait that has been associated with the dopaminergic system. These results suggest that white noise has no general effect on cognitive functions. Instead, they indicate differential effects on perception and cognition depending on a variety of factors such as task demands and timing of white noise presentation.

## Introduction

There is a growing body of research dealing with the sources and impact of noise in neural systems. Noise is an inherent feature of neural processing that affects perception, decision making, and motor function (Faisal et al., 2008) and is not necessarily detrimental but can also have constructive roles (McDonnell and Ward, 2011), for instance by improving reliability of spike firing in single neurons or boosting synchrony across neural populations (Ermentrout et al., 2008). Adding noise to a signal of interest can thus improve information processing – a mechanism that has been labeled stochastic resonance, or stochastic facilitation in a broader sense (Moss et al., 2004; McDonnell and Abbott, 2009; McDonnell and Ward, 2011).

Beneficial effects of noise have intensively been studied for sensory perception within the visual (Simonotto et al., 1997; Piana et al., 2000; Aihara et al., 2008; Schwarzkopf et al., 2011), somatosensory (Collins et al., 1996; Richardson et al., 1998; Linkenkaer-Hansen et al., 2004; Iliopoulos et al., 2014), and auditory domain (Zeng et al., 2000; Behnam and Zeng, 2003), across different modalities (Manjarrez et al., 2007; Lugo et al., 2008; Gleiss and Kayser, 2014), as well as for sensorimotor processing (Kitajo et al., 2003; Priplata et al., 2003; Wilkinson et al., 2008; Mendez-Balbuena et al., 2012; Trenado et al., 2014). Yet, diversity of conceptions and levels of observation render the underlying mechanisms difficult to grasp and demand cautious interpretation (Moss et al., 2004; McDonnell and Abbott, 2009; McDonnell and Ward, 2011).

Given that noise and its influence on neural processing is not limited to sensory signals but rather permeates every level of the nervous system (Faisal et al., 2008), stochastic facilitation should likewise be relevant for the implementation of higher cognitive functions. Indeed, computational modeling and cellular recordings of hippocampal sub-regions demonstrated the exploitation of noise in signal detection in hippocampal networks, indicating broad implications for memory formation and retrieval (Stacey and Durand, 2000; Yoshida et al., 2002).

Interestingly, experimental studies could also provide evidence for a modulation of higher cognitive functions through stimulation with external noise sources. For instance, noisy galvanic vestibular stimulation presented during recall of visual features of faces enhanced recall for these features (Wilkinson et al., 2008) and transcranial random noise stimulation over the motor cortex facilitated implicit motor learning (Terney et al., 2008). Acoustic noise has been shown to reduce errors in a delayed response task compared to music presentation and silence in monkeys (Carlson et al., 1997) and to affect the speed of arithmetical calculations in humans in an inverted-U shaped manner depending on loudness with reaction times (RTs) being shortest at an intermediate level of 77 dB (Usher and Feingold, 2000).

Differential effects of acoustic white noise on cognitive functions have been demonstrated for ADHD patients, children with severe attentional problems, and in a rat model of ADHD compared to controls, thereby hinting toward a mediating role of dopaminergic neuromodulation (Söderlund et al., 2007, 2010; Pålsson et al., 2011). In line with this notion, in a sample of healthy humans, white noise presented during encoding of scene images decreased sustained BOLD activity in the auditory cortex and substantia nigra/ventral tegmental area (SN/VTA) of the midbrain, and at the same time enhanced event-related effects of scene presentation in the same areas compared to a pure tone or no additional sound (Rausch et al., 2013). Recognition memory, however, was improved only slightly and inconsistently by white noise presentation (Rausch et al., 2013).

The SN/VTA is the origin of dopaminergic neurons projecting to different target sites such as the medial temporal lobe, striatum, and prefrontal cortex, which are key players in mnemonic processes and cognitive control (Düzel et al., 2009). A functional loop between SN/VTA and hippocampus has been suggested that controls the entry of information into long-term memory. Specifically, dopamine released phasically from the SN/VTA into the hippocampus is assumed to facilitate the entry of novel information, a process strongly influenced by stimulus salience and motivational state (Lisman and Grace, 2005). Consequently, encoding success and associated dopaminergic interactions of SN/VTA and hippocampus can be modulated by varying encoding incentive (Adcock et al., 2006; Schott et al., 2008). Gating and maintenance of memory representations on shorter timescales (i.e., working memory; Marié and Defer, 2003; Floresco and Magyar, 2006; Cools, 2011; Cools and D’Esposito, 2011) as well as attention allocation and salience assessment *per se* (Redgrave et al., 1999; Horvitz, 2000) have also been shown to depend on dopaminergic signaling.

The current study aimed to elucidate the effects of white noise on dopamine-dependent cognitive functions in a healthy sample, hypothesizing that white noise but not a pure tone presented via headphones would enhance mnemonic and attentional performance. Specifically, effects on working memory performance (Experiments 1–3), reward modulated long-term memory in a previously established reward incentive task (Adcock et al., 2006) (Experiment 4), and attentional (re-)orienting in the Posner task (Posner, 1980) (Experiment 5) were tested.

In Experiments 1–3, we expected white noise to improve the accuracy (and possibly also the speed) of working memory performance (Carlson et al., 1997). Times and duration of sound presentation were varied to further explore the temporal dynamics of such facilitation. During encoding in Experiment 4, we expected reward (Pleger et al., 2008) and white noise (Moss et al., 2004) to improve the accuracy and speed of perceptual judgments, whereas during recognition we expected improved memory performance for pictures that were encoded during white noise (Rausch et al., 2013) and with a high reward cue (Adcock et al., 2006). Furthermore, we wanted to explore the possibility that white noise interacts with reward value, which is also processed by the dopaminergic midbrain (Schott et al., 2008). Based on the fact that the SN/VTA strongly innervates the hippocampus, which in turn is especially involved in recollection based memory formation (Eichenbaum et al., 2007; Wittmann et al., 2007, 2005), we employed the remember/know procedure to distinguish between recollection and familiarity (Yonelinas, 2002). In Experiment 5, we expected faster and more accurate responses for validly cued targets (Posner, 1980), especially for highly reliable cues. Furthermore, we expected white noise to improve the accuracy and speed of target detection (Moss et al., 2004) and wanted to explore the possibility that white noise interacts with attention allocation which is influenced by midbrain dopamine, too (Horvitz, 2000).

Additionally, we assessed the personality traits novelty seeking and reward dependence in all experiments, as well as impulsivity in Experiments 4 and 5. While novelty seeking and reward dependence have been shown to be positively correlated with activity in the dopaminergic midbrain to novel and reward-predicting stimuli, respectively (Krebs et al., 2009) and dopaminergic stimulation in healthy subjects (Gerra et al., 2000) as well as Parkinson’s disease patients (Bódi et al., 2009), impulsivity scores have been linked to D2/D3 autoreceptor availability in the SN/VTA (Buckholtz et al., 2010). Our results suggest that white noise does not induce a general processing enhancement, but differentially affects perceptual and higher cognitive functions.

## Material And Methods

### Participants

In total, 167 healthy volunteers participated. Details on the number of subjects per experiment and their age and gender can be found in **Table 1**. Participants were required to be 18–35 years of age and not take any drugs. Participants were paid for participation (Experiments 1–3: 15 Euro; Experiment 4: performance-dependent 16–31 Euro, see section on Experiment 4; Experiment 5: 17 Euro).

**Table 1:** The number of subjects per experiment is given in total and separately per gender.

Experiment	N subjects (f/m)	Mean age (± SD)
1	40 (25/15)	25.0 (3.5)
2	41 (30/11)	25.3 (3.7)
3	42 (28/14)	25.2 (3.6)
4	19 (9/10)	27.0 (3.7)
5	25 (18/7)	25.0 (3.4)

*The mean age is given together with the standard deviation.*

### Stimuli Presentation

All visual stimuli were presented on an LCD monitor viewed from a distance of approximately 70 cm. Background color was gray. All auditory stimuli, i.e., the pure tone (500 Hz) and approximately Gaussian white noise with a flat spectrum over the range of audible frequencies between 20 Hz to 20 kHz, were administered at ∼70 dB via active noise canceling headphones (Bose QuietComfort^®^, Framingham, MA, USA). Importantly, this sound level was chosen based on previous studies showing enhanced mnemonic performance at similar levels (Usher and Feingold, 2000; Rausch et al., 2013). Unique noise samples were used for each single presentation and ear. All tasks were programmed and administered in MATLAB (The MathWorks Inc., Natick, MA, USA) using the Psychtoolbox-3 (Brainard, 1997; Kleiner et al., 2007).

### Questionnaires

All participants filled in a German version (Brändström et al., 2003) of the Temperament and Character Inventory Revised (TCI-R) with an adapted binary response format. Additionally, participants of Experiments 4 and 5 filled in a German version of the Barratt Impulsiveness Scale 11 (BIS-11) (for psychometric properties of this version see: Patton and Stanford, 1995). Mean values and standard deviations for the scales of interest: novelty seeking, reward dependence, exploratory excitability, and impulsivity can be found in **Table 2**. Furthermore, subjects indicated how pleasant they conceived the sounds on a 9 point scale (-4 to +4) subsequent to the experiment. The pleasantness rating was included based on findings linking subjective pleasantness with effectiveness of white noise in motor improvement (Trenado et al., 2014). Perceived pleasantness of white noise and pure tone did not differ in any sample. Similarly, collapsing both samples did not reveal a significant difference between pure tone [rating: -1.8 ± 1.4 (*SD*)] and white noise [rating: -1.2 ± 1.6 (*SD*); Experiment 4: *t*(18) = 1.04, *p* = 0.315, η^2^ = 0.056; Experiment 5: *t*(24) = 1.49, *p* = 0.150, η^2^= 0.084; collapsed: *t*(43) = 1.83, *p* = 0.075, η^2^ = 0.072].

**Table 2:** Mean values and standard deviations for novelty seeking, exploratory excitability, reward dependence, and impulsivity.

Experiment	Mean novelty seeking (±SD)	Mean exploratory excitability (±SD)	Mean reward dependence (±SD)	Mean impulsivity (±SD)
1	16.7 (5.8)	5.6 (2.4)	20.1 (3.8)	–
2	18.1 (5.3)	6.5 (2.0)	19.3 (5.9)	–
3	19.0 (6.0)	6.5 (2.1)	21.5 (5.0)	–
4	20.1 (5.4)	6.3 (2.2)	21.0 (4.1)	64.6 (6.5)
5	18.4 (5.0)	6.8 (1.4)	18.9 (4.5)	62.7 (10.4)

### Experiments 1–3

#### Task and Stimuli

**FIGURE 1:**
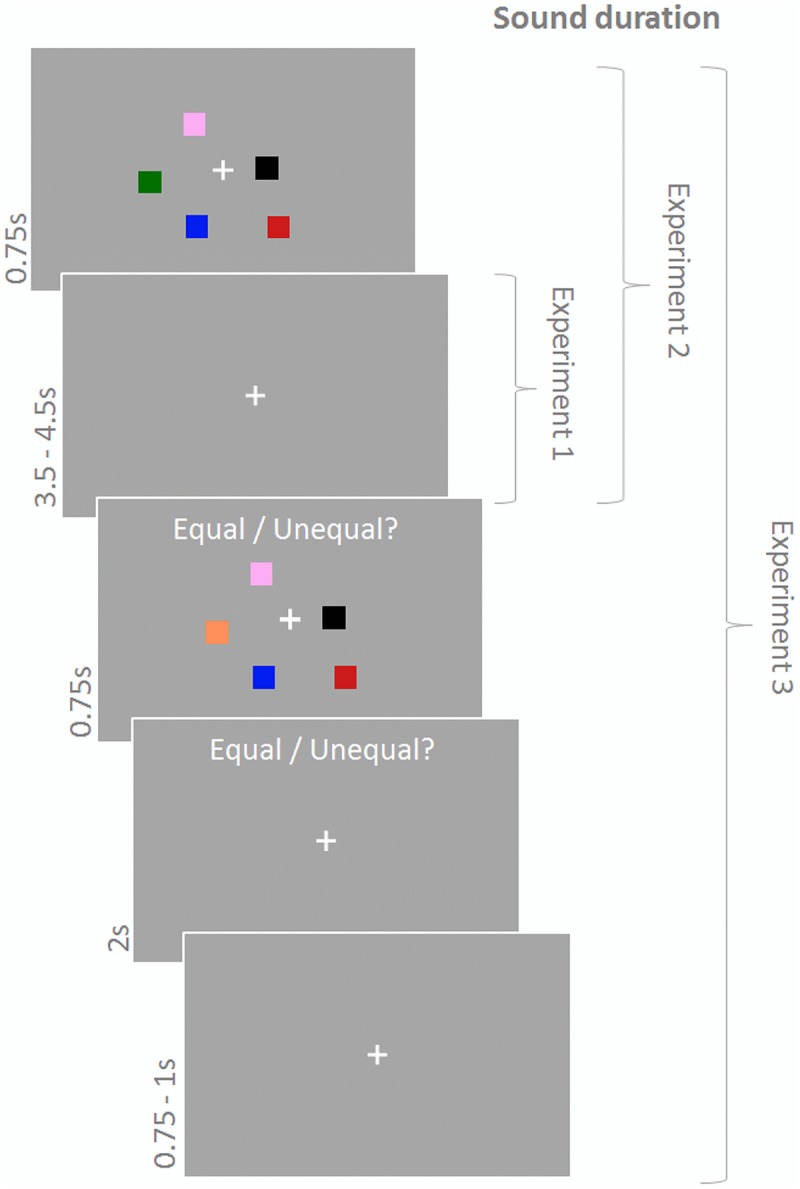
**Change detection task.** Participants had to indicate whether the test array differed from the memory array. Sound was presented during the maintenance period in Experiment 1, during encoding and maintenance in Experiment 2, and continously in Experiment 3.

In Experiments 1–3 participants performed a change detection paradigm (**Figure 1**) with memory arrays consisting of a central fixation cross and five differently colored squares. The memory array was presented for 750 ms and, after a delay of 3500–4500 ms (fixation cross), followed by the test array, which differed from the memory array on half of the trials with respect to the color of one of the squares. The test array was displayed for 750 ms and participants had to indicate via button press using their right index and middle finger whether memory array and test array were equal or not within 2750 ms from test array onset. A subsequent fixation cross was presented for 750–1000 ms until the next trial started. Squares (side length: 0.9°) were located randomly within a 4.3° × 4.3° area around the fixation cross with the constraint that Euclidian distance between the squares and between squares and center did not fall below 1.6°. Colors were randomly chosen without replacement out of a set of eight highly discriminable colors (yellow, green, white, orange, red, pink, blue, black). During the response period the text ‘Equal/Unequal?’ was written above the stimulus display.

Participants performed 150 trials in Experiments 1 and 2 and 180 trials in Experiment 3 (with an equal number of match and no-match trials). Importantly, experiments also differed in duration of sound presentation. In Experiment 1 sound was played during the delay period (maintenance) only, in Experiment 2 sound was played during maintenance and the memory array (encoding), and in Experiment 3 sound was played continuously. Furthermore, in Experiments 1 and 2 sound conditions were varied on a trial-by-trial basis whereas in Experiment 3 sound conditions were blocked for 30 trials (∼4 min). The number of trials was equal across sound conditions, the order of sound conditions was randomly chosen for each participant.

#### Data Analysis

Signal detection theory (Stanislaw and Todorov, 1999) was used to analyze accuracy: correct and incorrect trials without a change from test to memory array were defined as correct rejections (CRs) and false alarms (FA), whereas correct and incorrect change trials were defined as hits (H) and misses (M), respectively. The number (N) of the respective trials was used to calculate hit rates (HR = [NH + 0.5]/[NH + NM + 1]) and false alarm rates (FAR = [NFA + 0.5]/[NFA + NCR + 1]) for each condition. *d′* was then calculated by subtracting the inverse phi of the FAR from the inverse phi of the HR (*d′* = Φ^-1^[HR] - Φ^-1^[FAR]). The inverse phi maps probabilities onto *z*-scores according to the standard normal cumulative distribution function and thus transforms HR and FAR (probabilities) into the associated *z*-scores (number of standard deviations from the mean). The difference between these values is a measure of sensitivity/accuracy that is independent of response bias (Stanislaw and Todorov, 1999).

Reaction times were averaged for correct trials of each condition. Single trial values were only considered if they were within 1.5 times the interquartile range (IQR) above the upper quartile or below the lower quartile of the subjects’ responses in the respective condition and if they were not faster than 80 ms. This led to an exclusion of 4.0% of trials across all subjects and conditions.

Mean RT and *d′* values were entered into one-way repeated measures analyses of variance (ANOVAs) by sound condition (pure tone, white noise, no sound). Greenhouse–Geisser correction was used whenever the sphericity assumption was violated (*p* < 0.05). Planned *t*-tests between sound conditions (white noise vs. pure tone; white noise vs. no sound; pure tone vs. no sound) were considered significant at a Bonferroni corrected threshold of α = 0.05/3 = 0.017.

To evaluate the influence of the personality traits, novelty seeking and reward dependence on sound effects, personality scores were correlated with white noise benefit for accuracy and RTs [*d′*_(white noise)_ – *d′*_(no sound)_; RT_(no sound)_ – RT _(white noise)_]. For both variables larger values are associated with increased (faster/more accurate) performance. It should be noted here, that these variables can generally take on both, positive or negative values, depending on whether individual subjects benefit from white noise or not. Aggregated novelty seeking and reward dependence scores were used, as well as one novelty seeking subscale: exploratory excitability has been shown to be related to event-related responses in the SN/VTA (Krebs et al., 2009). Thus, the alpha level for correlations was Bonferroni corrected at α = 0.05/3 = 0.017.

### Experiment 4

#### Procedure

Experiment 4 was based on a previously established reward incentive task (Adcock et al., 2006) and divided in two parts: an encoding phase during which participants learned a set of indoor and outdoor scene images and a recognition test administered with a time lag of 40 min. Participants filled in the personality questionnaires while they waited for the second part to start. Sound was administered during the encoding phase only. Images in the encoding phase were associated with either high (2 Euro) or low (20 cent) incentive for correctly recognizing the respective picture in the recognition test. A cue preceding each image in the encoding phase informed participants about the reward at stake. In the recognition test, participants lost 1 Euro for each image that was erroneously classified as being old, in order to prevent them from trying to maximize their reward by classifying all images as old. After completing the recognition test participants were paid depending on their memory performance. They received 16 Euro plus 7.5% of what they gained during the recognition test (maximally 15 Euro on top).

#### Encoding Phase

**FIGURE 2:**
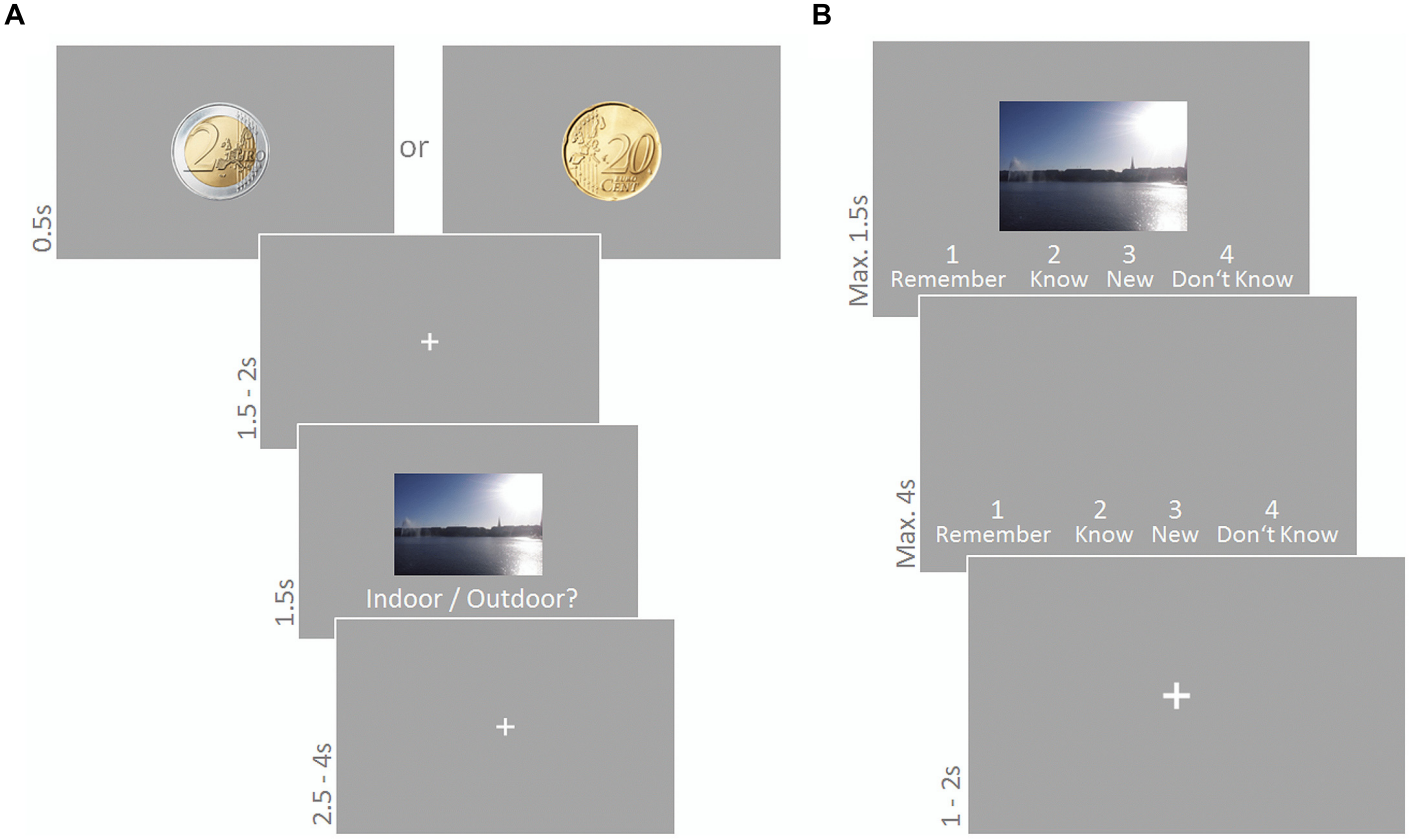
**Encoding phase **(A)** and recognition phase **(B)** of the reward based long-term memory task.** Sound was presented during the encoding phase only. **(A)** A cue indicated whether participants would receive high or low reward for correctly recognizing the following picture in the subsequent recognition test. They had to indicate indoor/outdoor status of the scene image. **(B)** Participants judged old/new status in a modified remember/know task.

Participants were presented 180 scene images (90 indoor, 90 outdoor; 6.5° × 10.7°) and had to indicate the indoor/outdoor status via button press using their right index and middle finger. Each trial started with a cue (2 Euro or 20 cent coin (90 times each); diameter 4.3°) presented for 500 ms informing the subject about how much money they could win for correctly recognizing the following picture in a subsequent recognition test. A fixation cross was presented for 1500–2000 ms, followed by a scene image presented for 1500 ms. The text ‘Indoor/Outdoor?’ was written below the image. Participants’ responses were only considered if they were given during scene presentation, i.e., within a time period of 1500 ms. Scene presentation was followed by a fixation cross presented for 2500–4000 ms. A representative trial is depicted in **Figure 2A**. None of the scenes depicted human beings or human body parts in the foreground. Sound was presented continuously and sound conditions were blocked for 30 trials (3.5 min); reward conditions varied trial-wise. The order of stimuli as well as sound and reward conditions was randomly chosen for each participant.

#### Recognition Phase

The 180 scene images from the encoding phase were presented again intermixed with 90 new images. Participants had to indicate their old/new status via button press in a modified version of the ‘remember/know’ recognition task (Tulving, 1985). Scenes were presented for 1500 ms (or until a button press) together with the four response alternatives *Remember, Know, New, Don’t Know*, and corresponding keyboard numbers. If no response was given within this time, response alternatives remained on the screen for another 4000 ms or until the subject responded. This was followed by a fixation cross for 1000–2000 ms until the next trial started. A representative trial is depicted in **Figure 2B**. The order of stimuli was randomly chosen for each participant.

#### Data Analysis

For the encoding phase, RTs and accuracy were considered. RTs for indoor/outdoor discriminations were averaged for correctly classified pictures within each condition. Single trial values were only considered if they were within 1.5 times the IQR above the upper quartile or below the lower quartile of the subjects’ responses in the respective condition and if they were not faster than 80 ms. This led to an exclusion of 2.8% of trials across all subjects and conditions. Hit rates (HR) for indoor/outdoor discrimination were calculated as the number of correct responses divided by the number of trials per condition.

Regarding the subsequent recognition test, corrected hit rates were calculated based on the assumption that recollection and familiarity rely on independent processes and ‘know’ responses are given in the absence of recollection (Yonelinas and Jacoby, 1995; Yonelinas, 2002). Corrected recollection rates (CR) were calculated as the probability of making a ‘remember’ judgment to an old item (R), corrected for the probability of making a ‘remember’ judgment to a new item (FAR for ‘remember’ responses [Fa R]; CR = R – Fa R). Corrected familiarity rates (CF) were calculated as the probability of making a ‘know’ judgment to an old item (K), corrected for the probability of making a ‘know’ judgment to a new item (FAR for ‘know’ responses [Fa K]) and the fact that ‘know’ responses were given in the absence of recollection (CF = [K – Fa K] / [1 – CR]).

For the encoding phase, mean RTs and hit rates were entered into two-way repeated measures ANOVAs with sound condition (pure tone, white noise, no sound) and reward (low, high) as within-subject factors.

For the recognition phase, corrected hit rates (CR and CF) were entered into three-way repeated measures ANOVAs with memory process (recollection, familiarity), sound condition (pure tone, white noise, no sound) and reward (low, high) as within-subject factors.

Greenhouse–Geisser correction was used whenever the sphericity assumption was violated (*p* < 0.05). Planned *t*-tests between sound conditions were considered significant at a Bonferroni corrected threshold of α = 0.05/3 = 0.017.

Novelty seeking, exploratory excitability, reward dependence, impulsiveness, and white noise pleasantness scores were correlated with white noise benefit [HR_(white noise)_ – HR_(no sound)_; RT_(no sound)_ – RT _(white noise)_] averaged for all reward and memory process conditions with a Bonferroni corrected alpha level of α = 0.05/5 = 0.010. This means, white noise benefit was calculated separately for each condition (memory process × reward) and then averaged across all conditions.

### Experiment 5

#### Task and Stimuli

**FIGURE 3:**
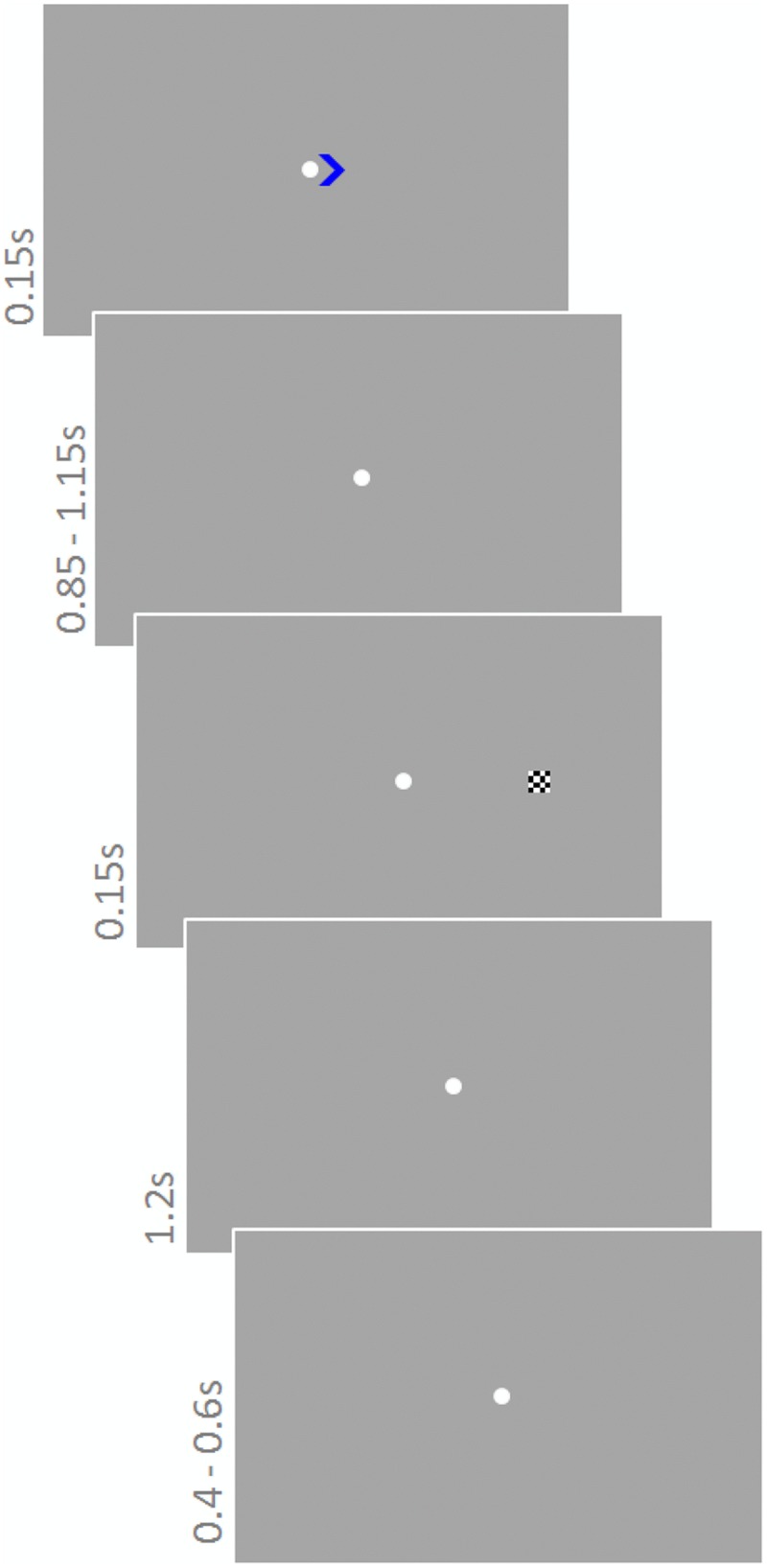
**Posner task.** A cue pointing to the left or right indicated where the upcoming target would appear. Cues were valid or invalid with different probabilities depending on the color of the cue (green or blue). One color signaled 80% validity, whereas the other signaled 65% validity. The task was to respond to the target as soon as it appeared with a ‘left’ or ‘right’ button press. Sound was presented continuously.

Participants performed a modified version of the Posner task (Posner, 1980) (**Figure 3**). They were instructed to constantly fixate on a white fixation point (diameter 0.3°) in the center of the screen. Each trial started with a colored cue (green or blue; 0.8 × 0.8°) pointing to the left or right (equal number of trials) indicating the upcoming target location. The cue was presented for 150 ms next to the fixation point. Depending on color, cues were valid or invalid with different probabilities. One color signaled 80% validity, whereas the other signaled 65% validity. Cue colors were counterbalanced across participants. After a delay of 850–1150 ms (fixation point), the target stimulus, a checkerboard (side length 0.5°) appeared for 150 ms on the left or right at an eccentricity of 5.4°. The response period lasted for 1200 ms and the next trial started after another 400–600 ms. Participants were instructed about the probability of the cues and asked to take this information into account during the 900 trials they performed. An additional 30 trials constituted catch trials, i.e., they did not contain a target stimulus but ended after cue presentation. These trials were not considered in the analysis. Sound was presented continuously and sound conditions were blocked for 62 trials (∼3 min); other conditions varied trial-wise. The order of cue directions and sound conditions was randomly chosen for each participant.

#### Data Analysis

The percentage of correct trials was calculated separately for each condition. RTs were averaged for correct trials within each condition. Again, trials were only considered if RT was within 1.5 the IQR above the upper quartile or below the lower quartile of the subjects’ responses in the respective condition and was not faster than 80 ms. This led to an exclusion of 5.1% of trials across all subjects and conditions.

Mean RTs and accuracy data were entered into three-way repeated measures ANOVAs with sound condition (pure tone, white noise, no sound), validity (valid, invalid), and probability (low, high) as within-subject factors. Greenhouse–Geisser correction was used whenever the sphericity assumption was violated (*p* < 0.05). Planned *t*-tests between sound conditions were considered significant at a Bonferroni corrected threshold of α = 0.05/3 = 0.017.

Novelty seeking, exploratory excitability, reward dependence, impulsiveness, and white noise pleasantness scores were correlated with white noise benefit [% correct responses _(white noise)_ – % correct responses _(no sound)_; RT_(no sound)_ – RT _(white noise)_] averaged across all probability and validity conditions with a Bonferroni corrected alpha level of α = 0.05/5 = 0.010. This means, white noise benefit was calculated separately for each condition (probability × validity) and then averaged across all conditions.

## Results

### Experiment 1

**FIGURE 4:**
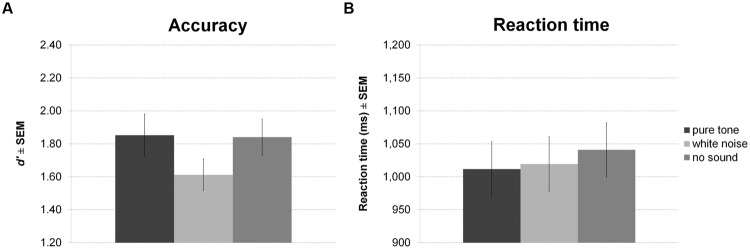
**Working memory task with sound during maintenance.** The figure depicts mean accuracy **(A)** and mean reaction time (RT) **(B)** together with the standard error of the mean (SEM) by sound condition in Experiment 1.

Sound presented in the delay period of the working memory task had a significant effect on accuracy (**Figure 4A**). However, in contrast to our expectation, participants were less accurate during white noise compared to no sound and pure tone. There was no difference between pure tone and no sound. Although showing a trend, the effect on RT was not significant (**Figure 4B**). Direct comparisons between sound conditions revealed slightly faster responses for pure tone compared to no sound, but this difference was significant only before correction for multiple comparisons.

There was no correlation of any of the personality scales with white noise benefit for accuracy or RT. All statistics for Experiment 1 are summarized in **Table 3**.

**Table 3:** Statistics for Experiments 1–3.

	F/t/r (df)	*p*	η^2^	ɛ
**Experiment 1**				
*Accuracy*				
Main effect: Sound	4.67 (2,78)	0.012*	0.107	-
No sound – white noise	3.07 (39)	0.004*	0.194	-
Pure tone – white noise	2.51 (39)	0.016*	0.139	-
Pure tone – no sound	0.12 (39)	0.906	<0.001	-
*Reaction time*				
Main effect: Sound	2.40 (2,78)	0.098	0.058	-
No sound – white noise	1.53 (39)	0.135	0.056	-
Pure tone – white noise	-0.58 (39)	0.568	0.008	-
Pure tone – no sound	-2.11 (39)	0.041	0.103	-
*Correlations: WNB accuracy*				
Novelty seeking	0.046	0.778	-	-
Exploratory excitability	-0.090	0.581	-	-
Reward dependence	0.076	0.640	-	-
*Correlations: WNB reaction time*				
Novelty seeking	0.193	0.232	-	-
Exploratory excitability	0.089	0.585	-	-
Reward dependence	-0.022	0.894	-	-
**Experiment 2**				
*Accuracy*				
Main effect: Sound	1.38 (2,80)	0.258	0.033	-
*Reaction time*				
Main effect: Sound	0.06 (2,80)	0.893	0.002	0.76
*Correlations: WNB accuracy*				
Novelty seeking	-0.101	0.529	-	-
Exploratory excitability	0.105	0.514	-	-
Reward dependence	-0.042	0.794	-	-
*Correlations: WNB reaction time*				
Novelty seeking	-0.280	0.077	-	-
Exploratory excitability	-0.184	0.250	-	-
Reward dependence	-0.058	0.718	-	-
**Experiment 3**				
*Accuracy*				
Main effect: Sound	0.55 (2,82)	0. 580	0.013	-
*Reaction time*				
Main effect: Sound	0.21 (2,82)	0.778	0.005	0.87
*Correlations: WNB accuracy*				
Novelty seeking	<0.001	>0.999	-	-
Exploratory excitability	0.196	0.214	-	-
Reward dependence	0.069	0.663	-	-
*Correlations: WNB reaction time*				
Novelty seeking	-0.293	0.059	-	-
Exploratory excitability	-0.174	0.270	-	-
Reward dependence	-0.255	0.103	-	-

*F-values for the ANOVA, *t*-values for direct comparisons and *r*-values for correlations are given. *p*-values are marked with an asterisk whenever considered significant. ɛ-values are given for Greenhouse–Geisser correction. WNB, white noise benefit.*

### Experiment 2

**FIGURE 5:**
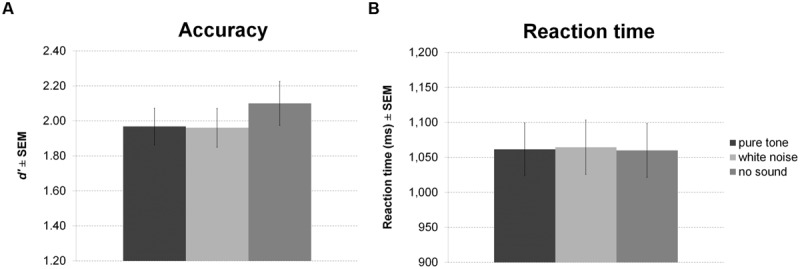
**Working memory task with sound during encoding and maintenance.** The figure depicts mean accuracy **(A)** and mean RT **(B)** together with the standard error of the mean (SEM) by sound condition in Experiment 2.

Sound presented during encoding and maintenance of the working memory task had no effect on accuracy (**Figure 5A**) or RT (**Figure 5B**).

No significant correlations of personality scales with white noise benefit were observed for accuracy or RT. All statistics for Experiment 2 are summarized in **Table 3**.

### Experiment 3

**FIGURE 6:**
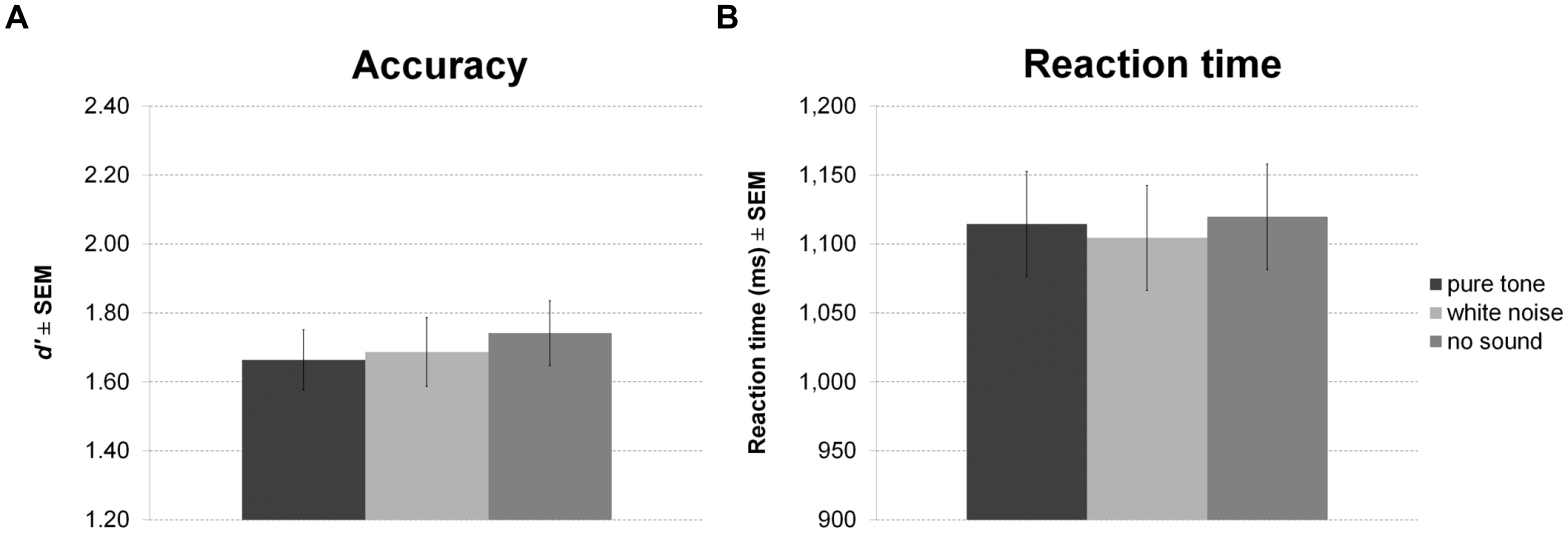
**Working memory task with continuous sound.** The figure depicts mean accuracy **(A)** and mean RT **(B)** together with the standard error of the mean (SEM) by sound condition in Experiment 3.

Again, there was no effect of sound condition on accuracy (**Figure 6A**) or RT (**Figure 6B**).

There were also no significant correlations of personality scales with white noise benefit for accuracy or RT. All statistics for Experiment 3 are summarized in **Table 3**.

### Experiment 4

**FIGURE 7:**
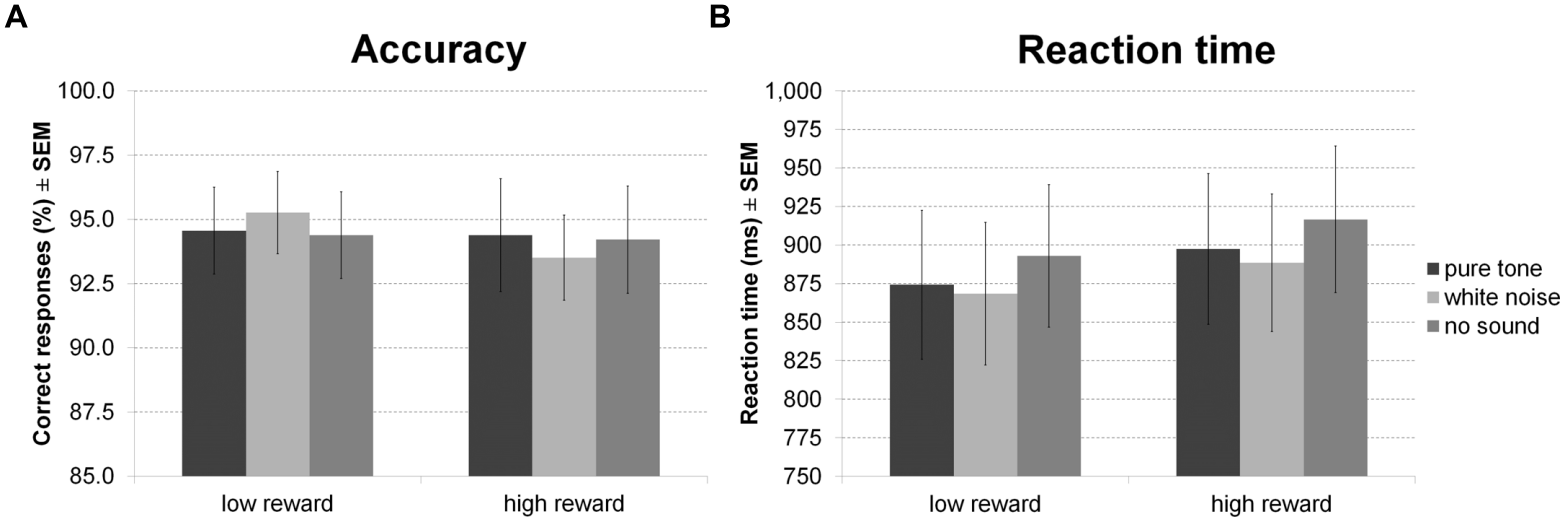
**Reward based long-term memory task: encoding.** The figure depicts mean accuracy **(A)** and mean RT **(B)** together with the standard error of the mean (SEM) during encoding by sound condition and reward in Experiment 4.

For accuracy of indoor/outdoor discrimination during encoding (**Figure 7A**) there was no main effect of sound or reward and no interaction between both factors. There was, however, a main effect of sound on RT (**Figure 7B**), in the absence of a main effect of reward or an interaction. Averaged across reward conditions, participants made faster indoor/outdoor judgments during white noise compared to no sound, while other differences were not significant.

**FIGURE 8:**
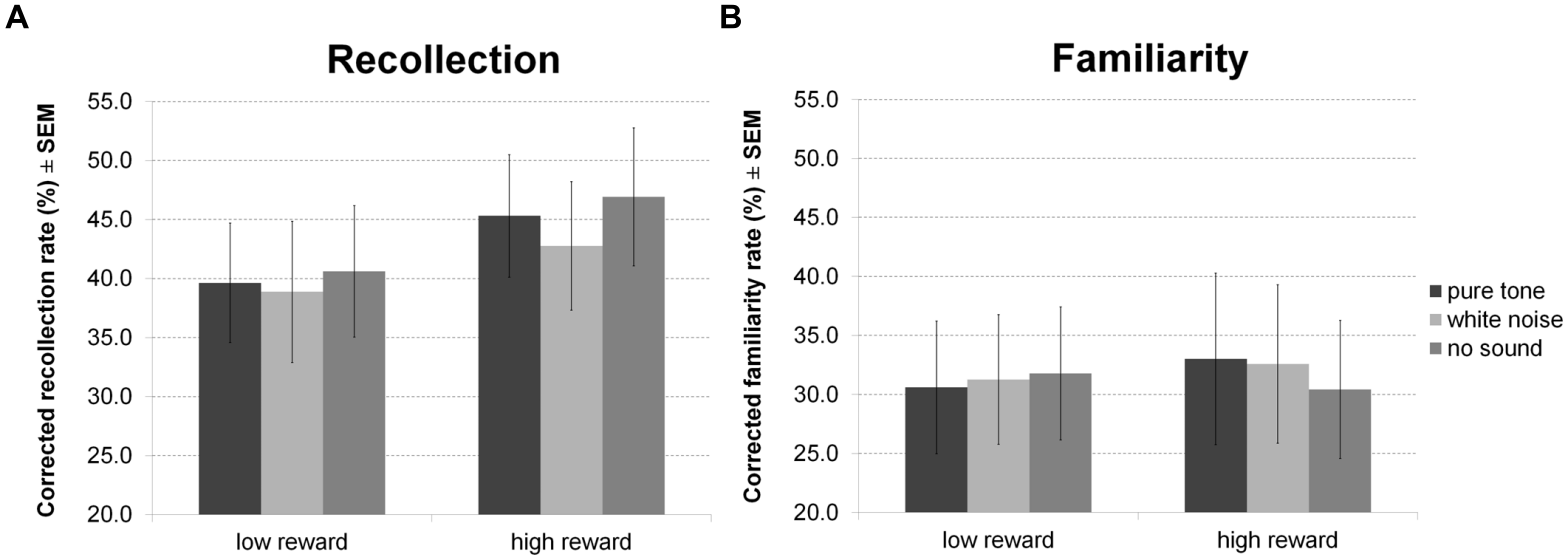
**Reward based long-term memory task: recognition.** The figure depicts mean corrected hit rates together with the standard error of the mean (SEM) during recognition by sound condition, reward, and memory process in Experiment 4.

For hit rates in the recognition memory test (**Figure 8**) there were no significant effects. However, separate analysis of recollection and familiarity revealed a main effect of reward for recollected but not familiar items, indicating correct recollection of relatively more high than low reward items. Other inferences remained equivalent (all *p* > 0.05).

There were no significant correlations of personality scales or white noise pleasantness with white noise benefit for RT during encoding, for accuracy during encoding, or for hit rates during recognition. All statistics for Experiment 4 are summarized in **Table 4**.

**Table 4:** Statistics for Experiment 4.

	F/t/r (df)	*p*	η^2^	ɛ
**Encoding**				
*Accuracy*				
Main effect: Sound	0.03 (2,36)	0.975	0.001	-
Main effect: Reward	0.81 (1,18)	0.380	0.043	-
Interaction: Sound x reward	0.62 (2,36)	0.543	0.033	-
*Reaction time*				
Main effect: Sound	3.40 (2,36)	0.044*	0.159	-
No sound – white noise	2.96 (18)	0.008*	0.328	-
Pure tone – white noise	0.81 (18)	0.428	0.035	-
No sound – pure tone	1.48 (18)	0.156	0.109	-
Main effect: Reward	3.01 (1,18)	0.100	0.143	-
Interaction: Sound × reward	0.02 (2,36)	0.953	0.001	0.75
*Correlations: WNB accuracy*				
Novelty seeking	0.077	0.755	-	-
Exploratory excitability	0.184	0.452	-	-
Reward dependence	0.009	0.970	-	-
Impulsivity	-0.219	0.369	-	-
Pleasantness	0.206	0.398	-	-
*Correlations: WNB reaction time*				
Novelty seeking	-0.267	0.268	-	-
Exploratory excitability	-0.450	0.053	-	-
Reward dependence	0.139	0.570	-	-
Impulsivity	-0.077	0.753	-	-
Pleasantness	-0.025	0.919	-	-
**Recognition**				
*Hit rates*				
Main effect: Memory	1.35 (1,18)	0.260	0.070	-
Main effect: Sound	0.10 (2,36)	0.905	0.005	-
Main effect: Reward	1.88 (1,18)	0.188	0.094	-
Interaction: Memory × sound	0.28 (2,36)	0.682	0.015	0.72
Interaction: Sound × reward	0.13 (2,36)	0.880	0.007	-
Interaction: Memory × reward	2.17 (1,18)	0.158	0.107	-
Simple effect: Reward for recollection	5.18 (1,18)	0.035*	0.223	-
Simple effect: Reward for familiarity	0.07 (1,18)	0.795	0.004	-
Interaction: Memory × sound × reward	0.61 (2,36)	0.550	0.033	-
*Correlations: WNB hit rates*				
Novelty seeking	0.031	0.899	-	-
Exploratory excitability	0.220	0.365	-	-
Reward dependence	0.069	0.777	-	-
Impulsivity	0.095	0.698	-	-
Pleasantness	0.245	0.313	-	-

*F-values for the ANOVA, *t*-values for direct comparisons and *r*-values for correlations are given. *p*-values a marked with an asterisk whenever they are considered significant. ɛ-values are given for Greenhouse–Geisser correction. WNB, white noise benefit.*

### Experiment 5

**FIGURE 9:**
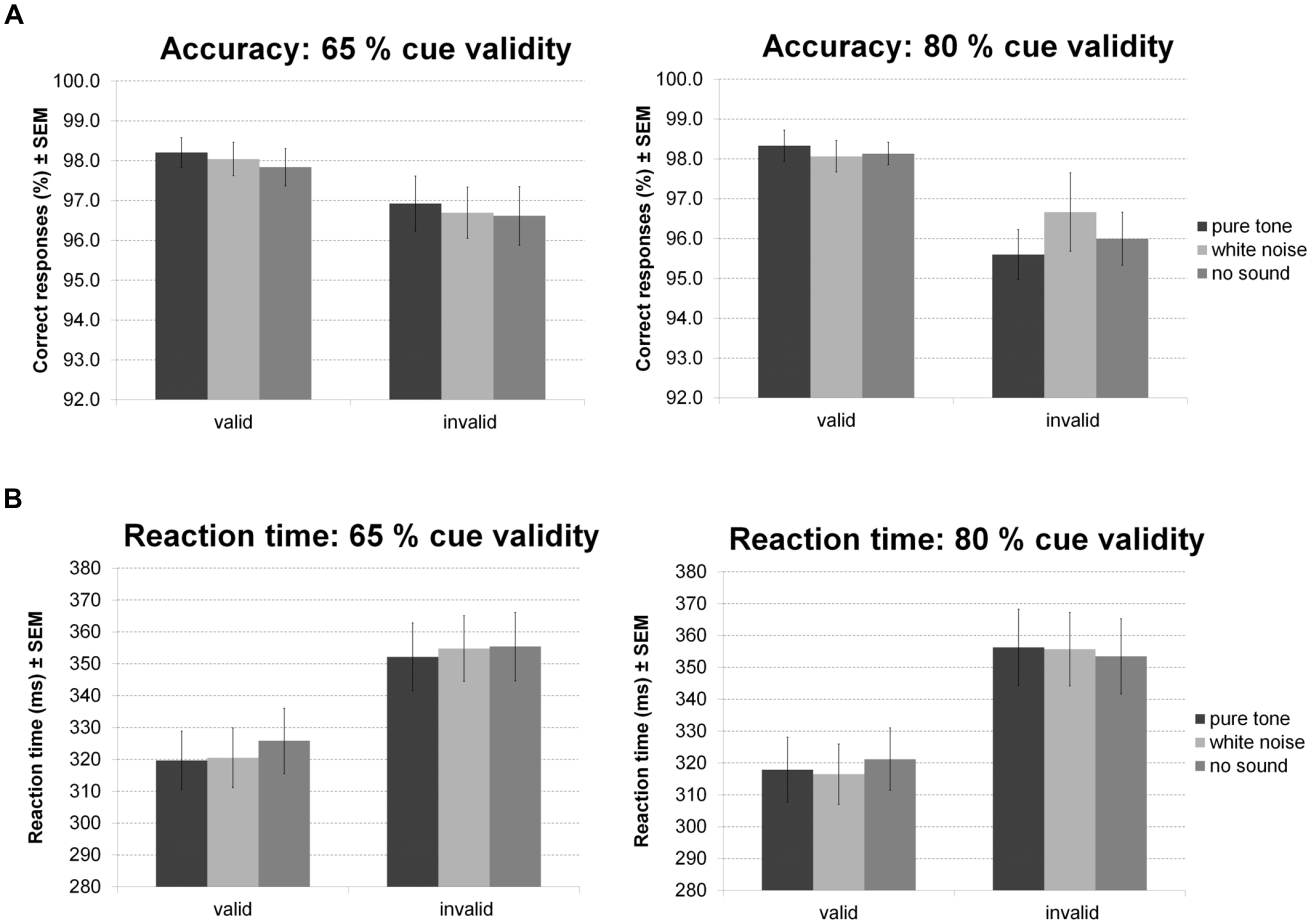
**Posner task.** The figure depicts mean accuracy **(A)** and mean RT **(B)** together with the standard error of the mean (SEM) by sound condition, validity, and probability in Experiment 5.

For accuracy during the Posner task (**Figure 9A**) there was a main effect of validity indicating more errors for invalid than valid trials. Furthermore, there was a trend for an interaction of validity and probability. Follow-up *t*-tests, however, revealed a strong validity effect on error rates within both probability conditions and no simple effect of probability in any validity condition. No other effect was significant.

Correspondingly, there was a main effect of validity on RT (**Figure 9B**): participants were faster during valid compared to invalid trials. There was also an interaction between validity and sound condition. However, none of the planned comparisons between sound conditions was significant for valid and invalid trials. Considering trends, white noise and pure tone similarly accelerated responses in valid but not invalid trials. There was no main effect of probability or sound and no interaction of probability and validity, probability and sound, or probability, validity, and sound.

**FIGURE 10:**
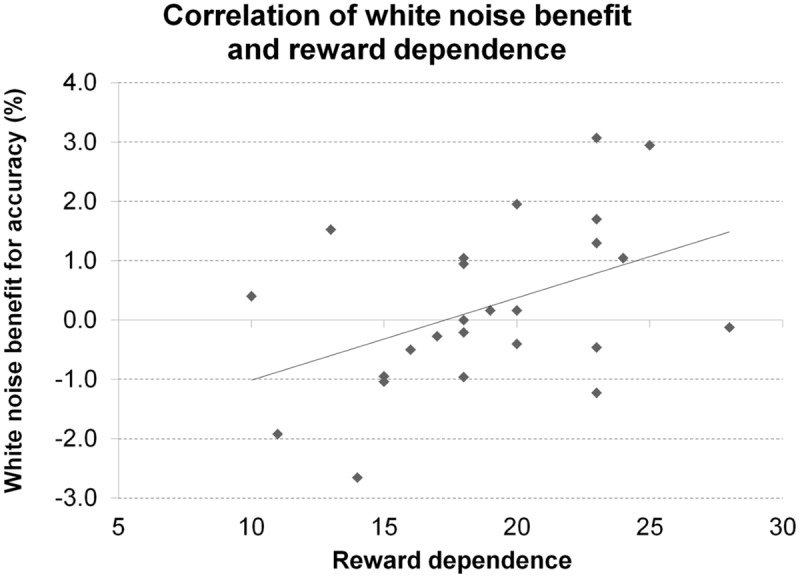
**Personality and performance in the Posner task.** The figure depicts the correlation of reward dependence with white noise benefit for accuracy in Experiment 5.

A significant correlation of reward dependence with white noise benefit for accuracy (**Figure 10**) did not survive correction for multiple comparisons. All other correlations were not significant even at an uncorrected threshold, both for accuracy and RT. All statistics for Experiment 5 are summarized in **Table 5**.

**Table 5:** Statistics for Experiment 5.

	F/t/r (df)	*p*	η^2^	ɛ
*Accuracy*				
Main effect: Validity	18.18 (1,24)	<0.001*	0.431	–
Main effect: Probability	0.77 (1,24)	0.388	0.031	–
Main effect: Sound	0.35 (2,48)	0.705	0.014	–
Interaction: Probability × sound	0.62 (2,48)	0.541	0.025	–
Interaction: Validity × sound	0.62 (2,48)	0.544	0.025	–
Interaction: Validity × probability	3.97 (1,24)	0.058	0.142	–
Simple effect: Validity for 65% probability	3.73 (24)	0.001*	0.367	–
Simple effect: Validity for 80% probability	3.98 (24)	0.001*	0.397	–
Simple effect: Probability for valid cues	0.69 (24)	0.497	0.020	–
Simple effect: Probability for invalid cues	1.47 (24)	0.155	0.083	–
Interaction: Validity × probability × sound	0.49 (2,48)	0.613	0.020	–
*Reaction time*				
Main effect: Validity	85.12 (1,24)	<0.001*	0.780	–
Main effect: Probability	0.96 (1,24)	0.336	0.039	–
Main effect: Sound	0.61 (2,48)	0.546	0.025	–
Interaction: Probability × sound	1.80 (2,48)	0.177	0.070	–
Interaction: Validity × sound	3.43 (2,48)	0.041*	0.125	–
Simple effect: Sound for valid cues				
Pure tone – white noise	0.13 (24)	0.895	<0.001	–
Pure tone – no sound	-2.05 (24)	0.052	0.149	–
White noise – no sound	-1.83 (24)	0.080	0.122	–
Simple effect: Sound for invalid cues				
Pure tone – white noise	-0.34 (24)	0.734	0.005	–
Pure tone – no sound	-0.09 (24)	0.928	<0.001	–
White noise – no sound	0.26 (24)	0.795	0.003	–
Interaction: Validity × probability	1.46 (1,24)	0.238	0.057	–
Interaction: Validity × probability × sound	0.20 (2,48)	0.823	0.008	–
*Correlations: WNB accuracy*				
Novelty seeking	0.086	0.683	–	–
Exploratory excitability	-0.019	0.927	–	–
Reward dependence	0.445	0.026	–	–
Impulsivity	-0.048	0.820	–	–
Pleasantness	0.311	0.130	–	–
*Correlations: WNB reaction time*				
Novelty seeking	0.016	0.940	–	–
Exploratory excitability	-0.319	0.120	-	–
Reward dependence	0.187	0.372	–	–
Impulsivity	-0.028	0.896	–	–
Pleasantness	0.089	0.674	–	–

*F-values for the ANOVA, *t*-values for direct comparisons and *r*-values for correlations are given. *p*-values a marked with an asterisk whenever considered significant. ɛ-values are given when Greenhouse–Geisser correction was used. WNB, white noise benefit.*

## Discussion

Previous work on noise benefits in neural processing and cognition has highlighted different, albeit not mutually exclusive, underlying mechanisms. Closely related to the physical concept of stochastic resonance (Gammaitoni et al., 1998), it has been stated that an optimal level of noise added to a subthreshold sensory signal can cause threshold crossing and thus enhance sensitivity for weak signals (Douglass et al., 1993; Collins et al., 1995; Wiesenfeld and Moss, 1995; Zeng et al., 2000; Moss et al., 2004). Moreover, noise has been linked to intra- and interregional neural synchronization (Moss et al., 2004; Ward et al., 2010). And finally, based on psychopathology research (Sikström and Söderlund, 2007), a link has been suggested between noise benefits and dopaminergic neuromodulation. By altering the ratio of tonic and phasic activity in the dopaminergic midbrain (i.e., SN/VTA) and its connectivity with higher cortical areas (i.e., superior temporal sulcus) (Rausch et al., 2013), externally applied noise might act on salience assessment (Redgrave et al., 1999; Horvitz, 2000), resource allocation (Boehler et al., 2011; Krebs et al., 2011), and cortical signal-to-noise ratio (Mattay et al., 1996, 2003; Li et al., 2001; Mattay et al., 2002; Winterer and Weinberger, 2004; Kroener et al., 2009). Other neurochemical systems such as GABA or norepinephrine, however, might also be involved (Coull et al., 2004; Samoudi et al., 2012).

The current study investigated the effects of acoustic white noise on attentional and mnemonic processes that strongly depend on dopaminergic signaling. Performance in a change detection task, a monetary incentive encoding task, and the Posner task was compared for concurrently presented white noise, a pure tone, and silence. Sound levels of 70dB were chosen based on previous studies showing an effectiveness of similar noise levels in improving mnemonic functions (Usher and Feingold, 2000; Rausch et al., 2013), although it should be noted, that some studies used slightly higher noise levels at around 75–80 dB (e.g., Carlson et al., 1997; Söderlund et al., 2010). Since, in the current study, pleasantness of pure tone and white noise were both rated slightly aversive on average (and strongly aversive by some subjects) already at 70 dB, we, however, suggest being careful when applying higher sound levels for an extended time period without individual adjustment. We hypothesized that white noise but not a pure tone would increase performance and that beneficial effects would correlate with personality dimensions known to be associated with interindividual differences in dopaminergic system parameters. These predictions could not be fully confirmed, leaving the modulatory influence of noise on higher cognitive functions a subject for further investigations.

### Experiments 1–3

Contrary to our expectation, working memory performance was impaired when white noise was presented in the delay period of a delay-match-to-sample task. This effect was selective for white noise and not a general effect of auditory stimulation, since the pure tone did not affect performance. This finding is in contrast to previously shown beneficial effects of white noise on working memory performance in monkeys (Carlson et al., 1997), but resembles deteriorating effects of white noise on memory for verbal sentences in healthy controls (Söderlund et al., 2007).

The systematic variation of sound presentation in Experiments 1–3 makes it possible to localize a time period and the associated cognitive function sensitive to acoustic noise. Participants were required to first encode the stimulus array, maintain a visuo-spatial representation during the delay and then match this sustained representation to the upcoming probe. All of these steps, i.e., encoding, maintenance, and decoding, are affected by noise and cognitive resources (Ma et al., 2014). In the current study, a negative effect of acoustic noise on accuracy was only observed when it was exclusively presented in the delay period. Thus, this effect cannot be caused by a direct influence on perception or matching processes during the stimulus display. Instead, noise either directly affects the population code sustained via interactions of prefrontal and association cortices during the maintenance phase (Sreenivasan et al., 2014), or it indirectly acts on the resources allocated to this process. The absence of this effect in Experiments 2 and 3 could be explained either by a compensatory beneficial effect on encoding or the necessity of white noise to set on during maintenance to be detrimental for performance.

Assuming a mediating role of dopamine, there are two likely explanations for an impairment rather than facilitation caused by white noise. First, an inverted-U shaped relationship has been used to describe behavioral performance as a function of dopamine (Vijayraghavan et al., 2007; Cools and D’Esposito, 2011) and noise levels (Usher and Feingold, 2000; Manjarrez et al., 2007; Sikström and Söderlund, 2007; Söderlund et al., 2007; Mendez-Balbuena et al., 2012; Trenado et al., 2014). Therefore, reduced accuracy in the working memory task can be explained by optimal baseline levels of dopamine or internal noise (Aihara et al., 2008) that may have been reduced by external white noise along the descending arm of the inverted-U shaped function leading to suboptimal performance.

Second, acoustic noise may have enhanced a different facet of working memory, than the one specifically required here. Depending on target site, dopamine has been implicated in different component processes of cognitive control and working memory: while stability and maintenance of information have been argued to be mediated by prefrontal dopamine receptors, flexibility and updating of working memory representations are likely controlled by striatal dopamine receptors (Cools et al., 2007; Cools and D’Esposito, 2011). The precise effect of dopamine on gating mechanisms in the striatum, however, remained debated: opening (Braver and Cohen, 2000; Badre, 2012; D’Ardenne et al., 2012) as well as locking (Gruber et al., 2006) the gate to working memory has been suggested as a consequence of phasic dopamine release from the SN/VTA. Changes in midbrain activity (Rausch et al., 2013) and putatively associated dopamine transmission caused by white noise administration might modulate cortico–striatal interactions in a way that improves updating at the cost of active maintenance of information, putting the system in a state of enhanced sensitivity to external stimulation and reduced stability of currently held representations in working memory. This would also be consistent with findings of enhanced connectivity between sensory and prefrontal brain areas during auditory noise stimulation (Ward et al., 2010) and noise benefits in sensory detection thresholds (Moss et al., 2004).

What argues against a relationship between dopamine and the detrimental effects of white noise in our working memory paradigm is the absence of a correlation with the dopamine mediated personality traits novelty seeking, exploratory excitability, and reward dependence. Therefore, these accounts remain speculative and need further empirical support. An alternative view is that our results are driven by changes in neurotransmitters other than dopamine (e.g., GABA or norepinephrine; see above) or unintended differences between sound conditions. Specifically, white noise has a more abrupt onset than a pure tone with a sinusoidal waveform, resulting in higher startle quality (Combs and Polich, 2006). This, in turn, might lead to a stronger disruption of ongoing encoding or maintenance processes when sound is turned on and off within a trial (as was the case in Experiments 1 and 2) as compared to a condition when it is presented continuously (as was the case in Experiment 3).

Finally, constant difficulty (i.e., working memory load) together with a dichotomous outcome measure (correct vs. incorrect) might result in ceiling effects for some subjects with high working memory capacity. Future studies could circumvent this issue to increase sensitivity by using a task with a parametric or continuous rather than binary outcome measure. This could for instance be the number of retained items out of a larger set of items or the accuracy of retained representations (e.g., continuous report of color or location).

### Experiment 4

Reward and white noise differently affected performance in the monetary incentive encoding task: while high potential monetary incentives enhanced recollective memory in the recognition phase, white noise accelerated the speed of perceptual judgments during encoding. An enhancing effect of recognition memory by monetary incentives was observed for recollection but not familiarity, yet, the interaction of incentive value and memory process failed to reach significance. This is in line with a previous study showing reward-driven gains in memory performance for high but not low confidence judgments (Adcock et al., 2006). Given that recollection should be associated with high confidence exclusively, whereas familiarity should reflect varying degrees of confidence (Yonelinas, 2002; Eichenbaum et al., 2007; Yonelinas and Parks, 2007) these results point in a similar direction.

White noise accelerated indoor/outdoor judgments during encoding as compared to silence, but did not affect subsequent recognition memory. This finding concurs with beneficial effects of noise on visual perception (Simonotto et al., 1997; Aihara et al., 2008; Schwarzkopf et al., 2011) and crossmodal stochastic resonance (Manjarrez et al., 2007; Lugo et al., 2008; Gleiss and Kayser, 2014). The current study extends these previous findings from low level signal detection to higher level visual category processing, which depends on lower and higher level visual and association areas along and in proximity to the ventral visual stream (Walther et al., 2009). As has been argued for sensory detection thresholds (Moss et al., 2004), externally applied white noise might boost sensory evidence for visual features toward a threshold for complex category decisions. Such a process could, however, be accomplished at every stage of visual processing, since higher level category processing strongly incorporates low level visual feature extraction (Renninger and Malik, 2004). Therefore, we cannot resolve whether an acceleration of indoor/outdoor judgments by white noise is due to a modulation of early visual processing exclusively or indicates that white noise also acts on category processing in higher visual areas directly.

An enhancement of (higher) sensory processing by white noise is also compatible with a mediating role of the dopaminergic system. For instance, white noise might affect the recruitment and allocation of attentional resources directed by the SN/VTA (Boehler et al., 2011; Krebs et al., 2011) or it might alter the gating of sensory stimuli controlled via cortico–striatal interactions (see above and specifically: Van Schouwenburg et al., 2010). Importantly, an acceleration of indoor/outdoor judgments has not been observed in the absence of reward (Rausch et al., 2013), which recruits the mesolimbic system (Adcock et al., 2006; Bunzeck et al., 2012). Although incentive value did not interact with noise benefit here, white noise might only modulate the speed of perceptual judgments in a context of high motivational state.

Beneficial effects of white noise on long term memory formation (Rausch et al., 2013) and retrieval (Usher and Feingold, 2000) have been reported previously for similar noise levels. However, in our current study, the increase in processing speed caused by white noise did not translate in superior memory formation for the respective pictures. Effects may be overall small in size and easily disrupted by contextual factors, such as motivational state and scanner environment (Rausch et al., 2013).

### Experiment 5

Performance in the Posner task was strongly dependent on cue validity. As expected, participants responded faster and more accurately on valid compared to invalid trials. This suggests successful orienting toward the cued location resulting in a processing advantage at that location (Posner, 1980; Posner et al., 1980; Doricchi et al., 2010; Petersen and Posner, 2012). A modulation of this effect by cue probability was at most subtle. Although such an interaction would be in line with assumptions about Bayesian integration in stimulus detection (Knill and Pouget, 2004) and a concrete model of uncertainty in a variant of the Posner task (Yu and Dayan, 2005), it has rarely been investigated empirically and led to inconsistent results (Jonides, 1980; Gottlob et al., 1999).

Participants responded marginally faster during auditory stimulation (for both white noise and pure tone) compared to silence on valid but not invalid trials, resulting in a stronger validity effect. This indicates enhanced processing at the cued location with no costs at the un-cued location. This pattern is inconsistent with faster basic sensorimotor processing (which should accelerate valid and invalid target detection) and a selective effect on orienting of attention (which should produce costs at the un-cued location). Instead, it might emerge if sound enhances two independent processes: one responsible for orienting toward the cued location and the other one responsible for reorienting in trials where no target appeared at that location, thereby counteracting costs at the un-cued location. This would be consistent with assumptions about two independent attention systems guided by dorsal and ventral parietal cortex responsible for orienting and reorienting, respectively (Fox et al., 2006; Corbetta et al., 2008; Vossel et al., 2012). Given the necessary difference in the number of trials in the valid and invalid condition, it is, however, also possible, that effects in invalid trials simply remained undiscovered due to higher error variance in the evaluation of within subject mean RT.

A significant correlation of the personality trait reward dependence with white noise benefit for accuracy did not survive correction for multiple comparisons. Since reward dependence has been linked with the dopaminergic system (Gerra et al., 2000; Krebs et al., 2009), this tentatively supports the claim for inter-individual differences in baseline dopamine levels to determine the effects of acoustic white noise on visual target detection but requires replication to be reasonably interpretable. Moreover, reward dependence has not only been linked to dopamine but also (and initially) to norepinephrine (Cloninger, 1985; Gerra et al., 2000; Ham et al., 2005) making it a rather unspecific marker for inter-individual baseline differences in dopamine levels.

## General Discussion

Taken together, we have shown that acoustic white noise selectively decreased working memory accuracy when presented during the delay period of a delay-match-to-sample-task, accelerated perceptual judgments for scene images, and left recognition memory for those images unaffected. The benefit in detection accuracy for cued visual targets caused by white noise weakly correlated with inter-individual differences in reward dependence. Finally, an unspecific effect of auditory stimulation on RTs was observed selectively for validly cued visual targets.

Opposing results for working memory accuracy and perceptual decisions (deterioration vs. enhancement of performance by white noise) indicate that the effects of acoustic white noise on cognitive functions do not rely on a broad and general processing enhancement but depend on the task at hand and associated cognitive demands. More specifically, there may be tasks that are facilitated by externally applied white noise (perceptual judgments) whereas others are impaired (working memory maintenance). As we have argued above, white noise might modulate midbrain activity (Rausch et al., 2013) and cortico–striatal interactions in a way that puts the system in a state of enhanced sensitivity to external stimulation and reduced stability of currently held representations, putatively by enhancing connectivity between sensory and prefrontal brain areas (Ward et al., 2010). Such an effect could be mediated by decreases in tonic and increases in phasic dopamine signaling from the SN/VTA to the striatum (Braver and Cohen, 2000; Badre, 2012; D’Ardenne et al., 2012; Rausch et al., 2013). This account would have been supported by a correlation of noise benefit with dopamine-dependent personality traits, which we only observed in the Posner task where white noise had no selective effect on performance. However, personality is only a very indirect marker for dopaminergic functioning that is quite possibly not sensitive enough to reveal the hypothesized correlation with underlying neuromodulatory variables. Furthermore, other trait variables not investigated here, such as attentiveness (Söderlund et al., 2010), might moderate the effects of noise on cognition.

Alternatively, the optimal level of white noise that facilitates rather than deteriorates performance might relate to the amount and precise nature of task demands. Other results might thus have been obtained with a sound level higher or lower than 70 dB. Moreover, inter- and intra-individual differences, for instance, concerning internal noise levels in relevant brain areas (Aihara et al., 2008) and/or baseline dopamine levels (Sikström and Söderlund, 2007) might moderate the effects. Testing this assumption would require a complex design including different noise levels, task difficulty levels, time points, and brain imaging techniques such as PET. Importantly, we suggest that such an experiment not only includes a noise condition but also a proper control sound (e.g., a pure tone or some meaningless correlated broad-band sound) to exclude unspecific effects of auditory stimulation.

## Conclusion

Acoustic noise does not exert a general processing enhancement on higher cognition. Instead, we demonstrated specific increases in the speed of high level perceptual judgments and decreases in the accuracy of maintained representations in working memory. These results encourage further research to focus more strongly on possible determinants of the direction of noise effects. This might include a thorough investigation of the influence of noise on different facets of cognitive control and sensory gating (i.e., flexibility versus stability) as well as state and trait parameters more directly associated with mesolimbic dopamine or other neurotransmitter systems.

## Author Contributions

NB and NH conceived the experiments and wrote the manuscript. NH performed the experiments and analyzed the data. NB provided supervision and funding.

## Ethics Statement

The study was conducted in Accordance with the Declaration of Helsinki and approved by the Ethics Committee of the Medical Council Hamburg. Participants gave written informed consent prior to the experiment.

## Conflict of Interest Statement

The authors declare that the research was conducted in the absence of any commercial or financial relationships that could be construed as a potential conflict of interest.
